# Clinical impact of butterbur shoot extract in dogs with oral melanoma: a combined phase 1 and 2 clinical trial

**DOI:** 10.1186/s12917-026-05326-w

**Published:** 2026-02-20

**Authors:** Shunsuke Noguchi, Masaru Furuya, Kenji Honda, Ryo Ito, Yukihiro Akao

**Affiliations:** 1 Veterinary Medical Center, School of Veterinary Science, Osaka Metropolitan University, 1-58 Rinku-Ourai kita, Izumisano, Osaka 598-8531 Japan; 2 Japan Animal Referral Medical Center, 3-14-7 Senba-Nishi, Minoo, Osaka 562- 0036 Japan; 3 Laboratory of Veterinary Internal Medicine, Graduate School of Veterinary Science, Osaka Metropolitan University, 1-58 Rinku-Ourai kita, Izumisano, Osaka 598-8531 Japan; 4 CCI Holdings Inc, 12 Shinhasama, Seki, Gifu, 501-3923 Japan; 5 The United Graduate School of Drug Discovery and Medical Information Sciences, Gifu University, 1-1 Yanagido, Gifu, 501-1193 Japan

**Keywords:** Canine melanoma, Clinical trial, Petasin, Petasites japonicus

## Abstract

**Background:**

Oral melanoma is one of the most devastating cancers in dogs. We previously developed a novel agent extracted from Japanese butterbur shoots and its petasin derivatives demonstrated anti-proliferative activity in canine oral melanoma cells. The aim of this study was to evaluate the safety and clinical efficacy of the butterbur shoot extract (BSE) in dogs with oral melanoma as a combined phase 1 and 2 study.

**Results:**

All enrolled dogs underwent radiation therapy or surgery as local therapy before BSE administration. We enrolled nine dogs in the phase 1 study, and BSE was orally administered at doses escalating from 50 to 200 mg/kg. Grade 1 alanine aminotransferase elevation and diarrhoea were observed in each dog. We determined that 200 mg/kg BSE was safe to administer. We subsequently administered it to sixteen dogs with stage 3 oral melanoma in the phase 2 study. Their progression-free survival and overall survival were compared with those of the historical controls, and adverse events were assessed. BSE administration significantly extended overall survival but did not prolong progression-free survival. No dose-limiting toxicities were observed.

**Conclusions:**

These results indicate that 200 mg/kg of BSE is safe to administer and may improve the outcome of canine oral melanoma.

**Supplementary Information:**

The online version contains supplementary material available at 10.1186/s12917-026-05326-w.

## Background

Melanoma is the most common malignancy of the canine oral cavity [1]. Oral melanoma has a high metastatic potential, which is associated with mortality. The clinical stage is the most valuable prognostic factor for dogs with oral melanoma. The prognosis of those with surgically resected oral melanoma based on clinical stage is as follow: stage 1, 874 days; stage 2, 818 days; and stage 3, 207 days [2]. Therefore, new therapeutic strategies are desired for advanced oral melanoma. The xenogeneic DNA vaccine targeting tyrosinase (Oncept^®^; Boehringer Ingelheim Pharmaceuticals, Ridgefield, CT, USA) has recently been applied to canine melanoma treatment [3]. However, reports on its clinical efficacy are conflicting [4–10]. Immune checkpoint inhibitors targeting programmed cell death 1 and programmed cell death 1-ligand 1 have been developed for canine oral melanoma and human melanoma, and their anti-tumour effects have been evaluated [11–15]. However, the response rates reported in these studies are insufficient.

Butterbur is a plant of the Asteraceae family distributed throughout Europe, Asia, and North America [16]. The butterbur herbal preparations are used to treat seasonal allergies and migraine [17, 18]. Butterbur extracts contain sesquiterpenes, pyrrolizidine alkaloids, flavonoids, phenolic acids, and volatile constituents. Sesquiterpenes, including petasin derivatives (petasin, isopetasin, neopetasin, S-petasin, iso-S-petasin, and S-neopetasin), are considered to be active components [16]. Several studies have reported that petasin exerts anti-proliferative effects by inhibiting the Akt/mammalian target of rapamycin in human colon cancer cells, activating the p53 pathway in mice and human melanoma cells, and inhibiting lipid metabolism [19–22]. More recently, it has been reported that petasin derivatives, including petasin, S-petasin, neopetasin, and neo-S-petasin extracted from butterbur (*Petasites japonicus*) shoot has anti-proliferative effects in a canine melanoma cell line. Petasin targets cancer cell-specific metabolic pathways via the inhibition of the mitochondrial electron transport chain complex 1 (ETCC1) [23]. Furthermore, petasin inhibits lung metastasis in mice models intravenously injected with B16F10 mouse melanoma cells. Thus, butterbur shoot extract (BSE), which includes petasin as a component, is a promising agent for melanoma treatment. Our unpublished preliminary data showed that BSE orally administered at a dose of 25–100 mg/kg does not have significant adverse events in healthy beagle dogs. Furthermore, we had assessed plasma level of petasin derivatives in a healthy beagle dog orally administered BSE at a dose of 100 mg/kg by High-Performance Liquid Chromatography-Photodiode Array. As a result, 1.8 µg/ml of petasin derivatives was detected at 4 h after BSE administration. The plasma level corresponded to 5.8 mM and was enough concentration to exhibit a cytotoxic effect in vitro [23].

The maximum tolerated dose (MTD) of BSE and its clinical impact were evaluated in the phase 1 and phase 2 studies, respectively. This study suggests that BSE, including petasin, is a promising therapy for canine oral melanoma.

## Methods

### BSE Preparation

Butterbur shoot was obtained from certain farms and a large-scale extraction was performed by the original equipment manufacturer, being able to sustain high quality of BSE. In addition, the quality of each lot was checked by high performance liquid chromatograph. And, quality of BSE tablets had been stable for one year at 4 ℃ and two months at room temperature (Additional file 1).

### Liquid chromatography-tandem mass spectrometry

Liquid chromatograph-tandem mass spectrometry (LC-MS/MS) experiments were performed to determine the concentration of petasin derivatives in BSE using an LCMS-8060NX (Shimadzu, Kyoto, Japan). The mobile phases consisted of 0.1% formic acid in water (Mobile Phase A) and 0.1% formic acid in acetonitrile (Mobile Phase B). The analyte was separated chromatographically using a Shim-pack Scepter C18-120 (2.1 × 50 mm, 3.0 μm) column (Shimadzu, Kyoto, Japan) with a constant flow rate of 0.2 mL/min. An LCMS-8060NX was used in positive mode with the multiple reaction monitoring modes (MRM), and the following mass transitions: petasin and neopetasin (m/z 317 > 105) and S-petasin and S-neopetasin (m/z 335 > 105).

### Study design and dogs

Client-owned dogs with naturally occurring oral melanoma that were treated with radiation therapy or surgical resection between March 1, 2020 and April 30, 2021, were enrolled in this open-label, dose-escalating phase 1 study via 3 + 3 design. Client-owned dogs with stage 3 oral melanoma that were treated with radiation therapy or surgical resection between January 1, 2021 and June 30, 2023, were enrolled in the current open-label single-arm phase 2 study. Three-dimensional conformal radiation therapy (3D-CRT) was performed using a linear accelerator with an X-ray energy output of 4 MV (Primus Mid Energy; Canon Medical Systems, Tochigi, Japan). The dose prescription was 8.0 Gy in four fractions weekly, for a total dose of 32 Gy. The gross tumour volume (GTV) was defined by the contrast-enhancing area on CT images and the affected side mandibular and medial retropharyngeal lymph nodes. The clinical target volume (CTV) was contoured from 0.3 to 0.8 cm of GTV to include regions at risk for microscopic disease. Then, the CTV margin was extended three dimensionally by 0.2 cm to define the planning target volume (PTV), accounting for safety margins for positioning errors. The isocentre and beam arrangements for each plan were determined on the basis of the location of the tumour and adjacent critical normal structures. Treatments were delivered in parallel in opposing fields or in four fields in multiple beam arrangements. Portal imaging was performed before the first treatment session to ensure appropriate patient positioning. Surgical resection was performed with a 1-cm lateral margin, and the lesion was removed with adjacent osseous tissue. A histological diagnosis was made for all dogs, and clinical staging was based on cytology of lymph node and computed tomography (CT) examination. The clinical stage was determined based on the World Health Organization (WHO) staging scheme for dogs with oral melanoma. This clinical study was approved by the Ethics Review Board of our institute (approval number: R2-001). Written owner consent was an enrolment criterion for all dogs in the study group. 3D-CRT and CT examination was performed under general anesthesia using intravenous injection of propofol (6 mg/kg, MSD Animal Health, Tokyo, Japan) and inhalational isoflurane (Bussan Animal Health, Osaka, Japan).

The client-owned dogs with stage 3 (primary lesion > 4 cm or metastasis to the regional lymph node) oral melanoma that were treated with 3D-CRT, which was performed by the above mentioned protocol, without adjuvant therapies between April 1, 2017 and June 30, 2023, were enrolled as the historical control group.

This clinical trial was designed according to animal research: reporting of in vivo experiments 2.0 guidelines [24].

### BSE treatment regimen

BSE tablets were provided by CCI Holdings Inc. (Gifu, Japan). The tablet sizes (diameter) were 7.5, 9, 10 mm, which contained 100, 150, and 200 mg BSE, respectively. Oral administration of BSE was started 14 days after the surgical treatment or completion of 3D-CRT. In the phase 1 study, the starting dose of BSE was 50 or 100 mg/kg per day, which was escalated by 50 mg/kg to 200 mg/kg every 2 weeks. In the phase 2 study, BSE was administered at a dose of 200 mg/kg per day from 14 days after the local therapy. Several dogs participated in phase 2 study after the phase 1 study and were administered BSE until the owner decided to discontinue. While the dogs were administered BSE, any other cytotoxic drugs including cox-2 inhibitors were not administered.

### Clinical assessments

All enrolled dogs were evaluated for adverse events (AEs). Complete blood cell count, blood biochemistry, and urinary analysis were performed every 2 weeks during the phase 1 study. For the phase 2 study, these assessments were performed every 2 weeks until 8 weeks and then every 8 weeks. AEs were assessed according to the Veterinary Cooperative Oncology Group-Common Terminology Criteria for Adverse Events (VCOG-CTCAE) v2 [25].

Adequate local control (ALC), which was evaluated at the start of BSE administration, was defined as complete and partial response after local therapy according to the Response Evaluation Criteria for Solid Tumours in Dogs (v1.0) [26]. The response to BSE was evaluated by radiographic or CT examination every 2 weeks. Target lesions were defined as the primary lesion, regional lymph nodes, and pulmonary nodules. Overall survival (OS) was defined as the duration from the start of local therapy to death. Progression-free survival (PFS) was defined as the duration from the start of local therapy to regrowth of the primary lesion or appearance of pulmonary nodules.

### Statistical analysis

The characteristics of the groups were compared using the Chi-squared test in Excel software. OS and PFS were calculated, and significant differences in the survival duration between BSE and control groups were analysed using log-rank tests with GraphPad Prism software (USACO, Tokyo, Japan). In addition, survival differences were evaluated between BSE excluding the dogs treated with surgical resection and control groups. The associations between potential variables predicting BSE efficiency were assessed using Cox proportional hazards models with EZR software (https://www.jichi.ac.jp/usr/hema/EZR/statmed.html). The dogs were censored when lost to follow-up. Statistical significance was set at *p* < 0.05.

## Results

### Petasin content in BSE

**Fig. 1:**
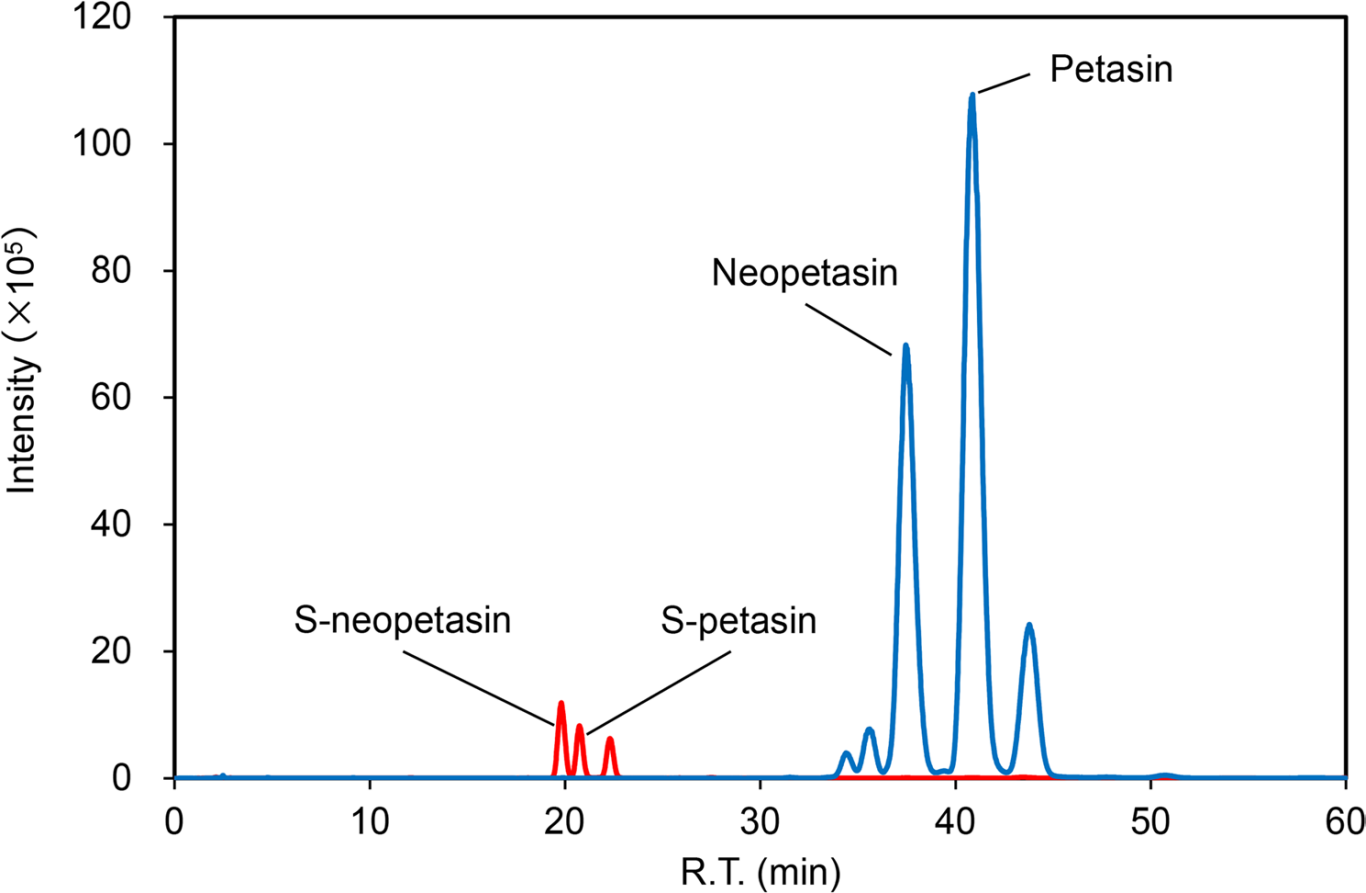
MS chromatogram for BSE. R.T., retention time

LC-MS/MS detected the petasin derivatives in BSE, including petasin (67.7 µg/mg), neopetasin (19.0 µg/mg), S-neopetasin (5.3 µg/m), and S-petasin (2.5 µg/mg) (Fig. 1). Petasin was the most abundant component among the petasin derivatives in BSE.

### Toxicities of BSE in phase 1 study

**Fig. 2:**
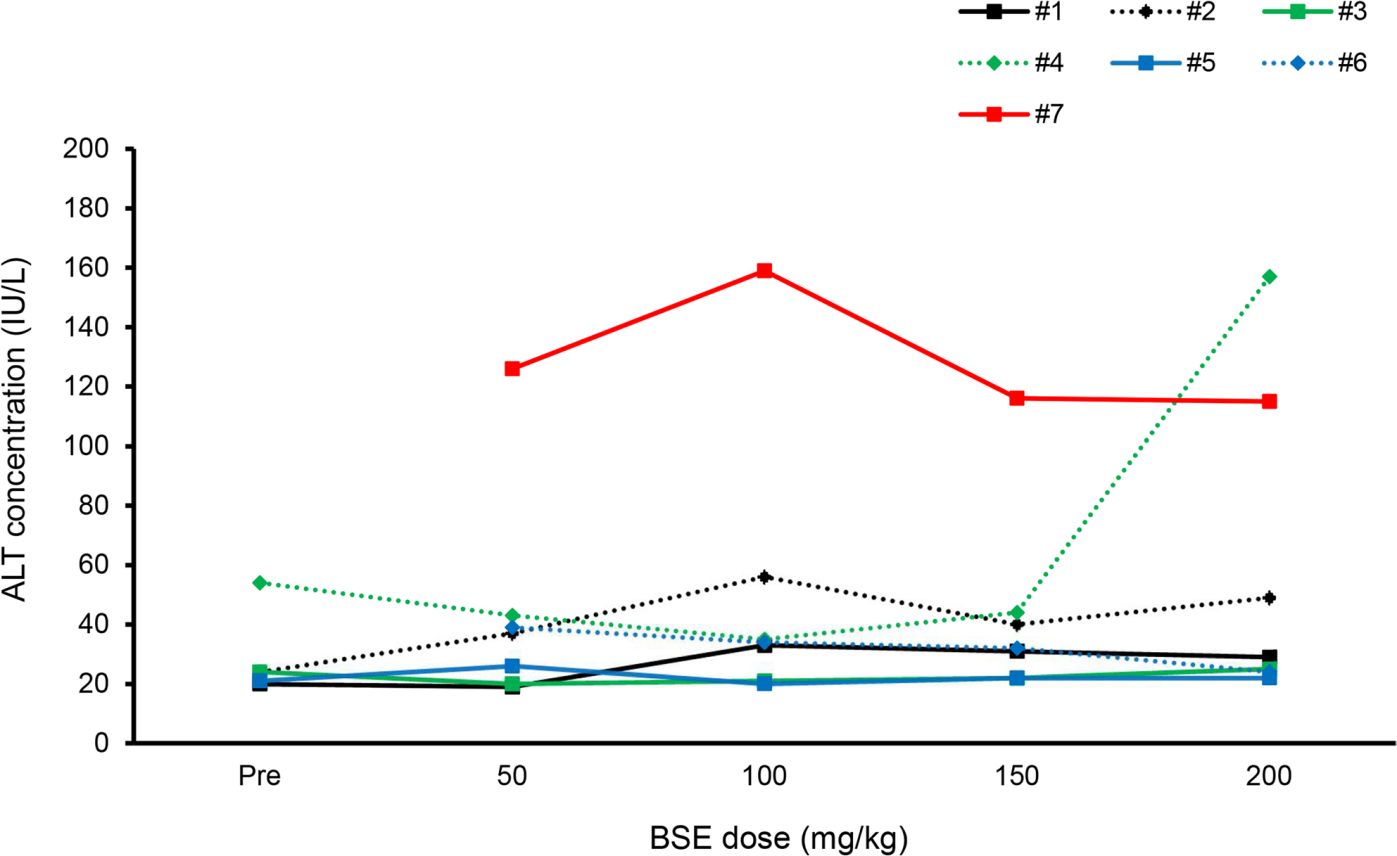
Time-course change in the ALT concentration in the phase 1 study. Grade 1 elevation was observed in dog #4

Nine dogs were enrolled in the phase 1 study. Their characteristics are summarized in Table 1. Two of the nine dogs (dogs 1 and 6) were withdrawn after receiving the 100 mg/kg dose, because their owners could not administer BSE due to the high number of tablets. Mild AEs were observed in two dogs: grade 1 elevation of alanine aminotransferase (ALT) concentration in dog #4 (Fig. 2) and grade 1 diarrhoea in dog #7. Urinalysis did not reveal any abnormal changes in all dogs tested. These results indicate that 200 mg/kg/day is the highest feasible dose based on tablet size available and its dose of BSE is well tolerated in dogs with oral melanoma.

**Table 1:** The characteristics of the dogs enrolled in phase 1 study

Patient #	Breed	Sex	Age (y)	B.W. (kg)	Clinical stage	Starting dose (mg/kg)	AEs
1	MD	CM	12	9.3	3	50	None
2	MD	CM	16	7.1	3	50	None
3	TP	F	14	2.8	2	50	None
4	MD	SF	14	4.1	2	50	ALT elevation (G1)
5	ACS	SF	13	10.5	3	50	None
6	MD	M	9	5.8	3	50	None
7	TP	SF	15	4	3	50	Transient diarrhoea (G1)
8	MD	F	9	4.4	3	100	None
9	MD	SF	13	4.2	3	100	None

*MD* Miniature Dachshund, *TP* Toy Poodle, *ACS* American Cocker Spaniel﻿, *CM* Castrated male, *M* Male, *F* Female, *SF* Spayed female, *ALT* Alanine aminotransferase

### Effect of BSE in phase 2 study

Sixteen dogs including five that participated in the phase 1 study, were enrolled in the phase 2 study. We assessed the effect of BSE at a dose of 200 mg/kg/day on PFS and OS. The characteristics of the dogs are summarized in Table 2. Macroscopic lesions were observed in all of the dogs other than #3 and 5 cases treated with surgical treatment, when BSE administration was started. In contrast, 25 dogs with stage 3 oral melanoma were enrolled as the historical control group. The characteristics of these dogs are summarized in Table 3. Comparison of clinical characteristics between the BSE and control groups did not yield any significant differences (Table 4).

**Table 2:** The characteristics of the dogs enrolled in phase 2 study

Patient #	Breed	Sex	Age (y)	B.W. (kg)	Primary lesion	Lymphnode metastasis	Bone lysis	Starting dose (mg/kg)	AEs	Primary treatment	Surgical margin	ALC	PFS (day)	OS (day)
1	MD	CM	16	7.1	Maxilla	No	No	50	None	RT	N.A	Yes	148	342
2	ACS	SF	13	10.5	Mandibule	Yes	No	50	None	RT	N.A.	Yes	57	96
3	TP	SF	15	4.0	Mandibule	Yes	Yes	50	Diarrhea (G1)	Surg.	Complete	Yes	596	612
4	MD	F	9	4.4	Maxilla	Yes	Yes	100	None	RT	N.A.	Yes	35	110
5	MD	SF	13	4.2	Maxilla	Yes	Yes	100	None	Surg.	Complete	Yes	305	335
6	TP	SF	13	3.2	Mandibule	Yes	Yes	150	None	RT	N.A.	Yes	351	1189
7	MD	CM	17	6.8	Mandibule	No	Yes	150	None	RT	N.A.	Yes	105	157
8	Mongrel	SF	12	3.4	Mandibule	Yes	Yes	200	None	RT	N.A.	No	56	98
9	MD	SF	13	4.9	Hard palate	Yes	No	200	None	RT	N.A.	No	615	726
10	MD	CM	13	5.5	Hard palate	No	Yes	200	None	RT	N.A.	Yes	43	97
11	TP	SF	13	3.6	Maxilla	Yes	Yes	200	None	RT	N.A.	Yes	75	119
12	TP	SF	17	8.2	Maxilla	Yes	Yes	200	None	RT	N.A.	No	32	103
13	Shih Tzu	CM	15	6.0	Mandibule	No	Yes	200	None	RT	N.A.	No	60	146
14	TP	CM	18	5.2	Mandibule	No	Yes	200	None	RT	N.A.	No	170	724
15	MD	CM	15	6.9	Mandibule	No	Yes	200	None	RT	N.A.	No	118	155
16	Mongrel	SF	13	4.0	Hard palate	No	No	200	None	Surg.	Incomplete	Yes	140	215

*MD* Miniature Dachshund, *TP* Toy Poodle, *ACS* American Cocker Spaniel, *CM* Castrated male, *F* Female, *SF* Spayed female, *AEs* Adverse events, *RT* Radiation therapy, *Surg* Surgical treatment, *N.A* Not applicable, *ALC* Adequate local control

**Table 3:** The characteristics of the dogs enrolled in phase 2 study as a historical control group

Patient #	Breed	Sex	Age (y)	B.W. (kg)	Primary lesion	Lymphode metastasis	Bone lysis	Primary treatment	ALC	PFS (day)	OS (day)
1	MD	CM	14	7.2	Maxilla	No	Yes	RT	No	128	128
2	YT	SF	14	3.5	Maxilla	No	Yes	RT	Yes	175	202
3	Mongrel	CM	15	6.4	Mandibule	Yes	No	RT	Yes	189	354
4	Pekingese	CM	15	3.7	Maxilla	No	Yes	RT	No	50	97
5	Papillon	SF	13	2.4	Maxilla	No	Yes	RT	No	83	119
6	MD	CM	13	3.2	Maxilla	No	No	RT	Yes	159	386
7	MD	CM	18	7.5	Mandibule	Yes	Yes	RT	Yes	29	90
8	Pekingese	SF	12	6.3	Maxilla	Yes	Yes	RT	No	22	30
9	MD	M	13	4.7	Mandibule	No	Yes	RT	Yes	27	41
10	MD	SF	15	3.2	Mandibule	No	Yes	RT	Yes	113	150
11	TP	CM	13	4.0	Mandibule	No	Yes	RT	No	22	50
12	MD	CM	13	5.3	Mandibule	No	Yes	RT	Yes	126	126
13	Mongrel	M	9	4.5	Soft palate	No	No	RT	Yes	71	259
14	TP	CM	13	4.4	Maxilla	Yes	Yes	RT	Yes	61	104
15	TP	SF	10	1.7	Maxilla	No	Yes	RT	Yes	87	87
16	Pumi	SF	12	7.5	Maxilla	Yes	Yes	RT	Yes	315	371
17	MD	SF	12	6.0	Soft palate	No	No	RT	Yes	228	228
18	MD	SF	13	5.4	Soft palate	No	No	RT	No	107	142
19	Basenji	M	10	10.0	Mandibule	No	Yes	RT	Yes	34	46
20	Papillon	CM	15	3.5	Mandibule	Yes	No	RT	No	21	61
21	Pomeranian	CM	12	3.4	Maxilla	No	Yes	RT	No	48	145
22	Shiba	CM	12	8.5	Maxilla	No	Yes	RT	Yes	147	200
23	MD	CM	13	7.0	Maxilla	Yes	Yes	RT	Yes	48	80
24	CKCS	CM	10	12.3	Maxilla	No	Yes	RT	No	29	92
25	Pomeranian	SF	12	4.9	Mandibule	Yes	Yes	RT	Yes	23	68

*MD* Miniature Dachshund, *TP* Toy Poodle, *ACS* American Cocker Spaniel, *CM* Castrated male, *F* Female, *SF* Spayed female

**Table 4:** Comparison of clinical characteristics between BSE and control group

		BSE	Control	*p value*
Sex (%)	male	6 (37.5)	16 (64)	0.181
	female	10 (62.5)	9 (36)	
Age (y)		13	13	0.259
Body weight (kg)		4.4	4.2	0.964
Locaiton (%)	maxilla	5 (31.3)	13 (52)	0.423
	mandibule	8 (50)	9 (36)	
	others	3 (18.7)	3 (12)	
Lymphode metastasis (%)	yes	7 (43.8)	8 (32)	0.667
	no	9 (56.2)	17 (68)	
Bone lysis (%)	yes	12 (75)	19 (76)	0.764
	no	4 (25)	6 (24)	
ALC (%)	yes	10 (62.5)	16 (64)	0.814
	no	6 (37.5)	9 (36)	

*BSE* butterbur shoot extract, *ALC* Adequate local control

Dose-limiting AEs were not observed in any of the dogs administered a dose of 200 mg/kg, although transient grade 1 gastrointestinal disorder was observed in #3 dog. Five dogs were withdrawn from the phase 2 study because of their worsening general condition due to tumour progression (#2, 8, 10, and 11 cases) or their owners could not administer the tablets (#15 case). The five dogs were subsequently lost to follow-up.

**Fig. 3:**
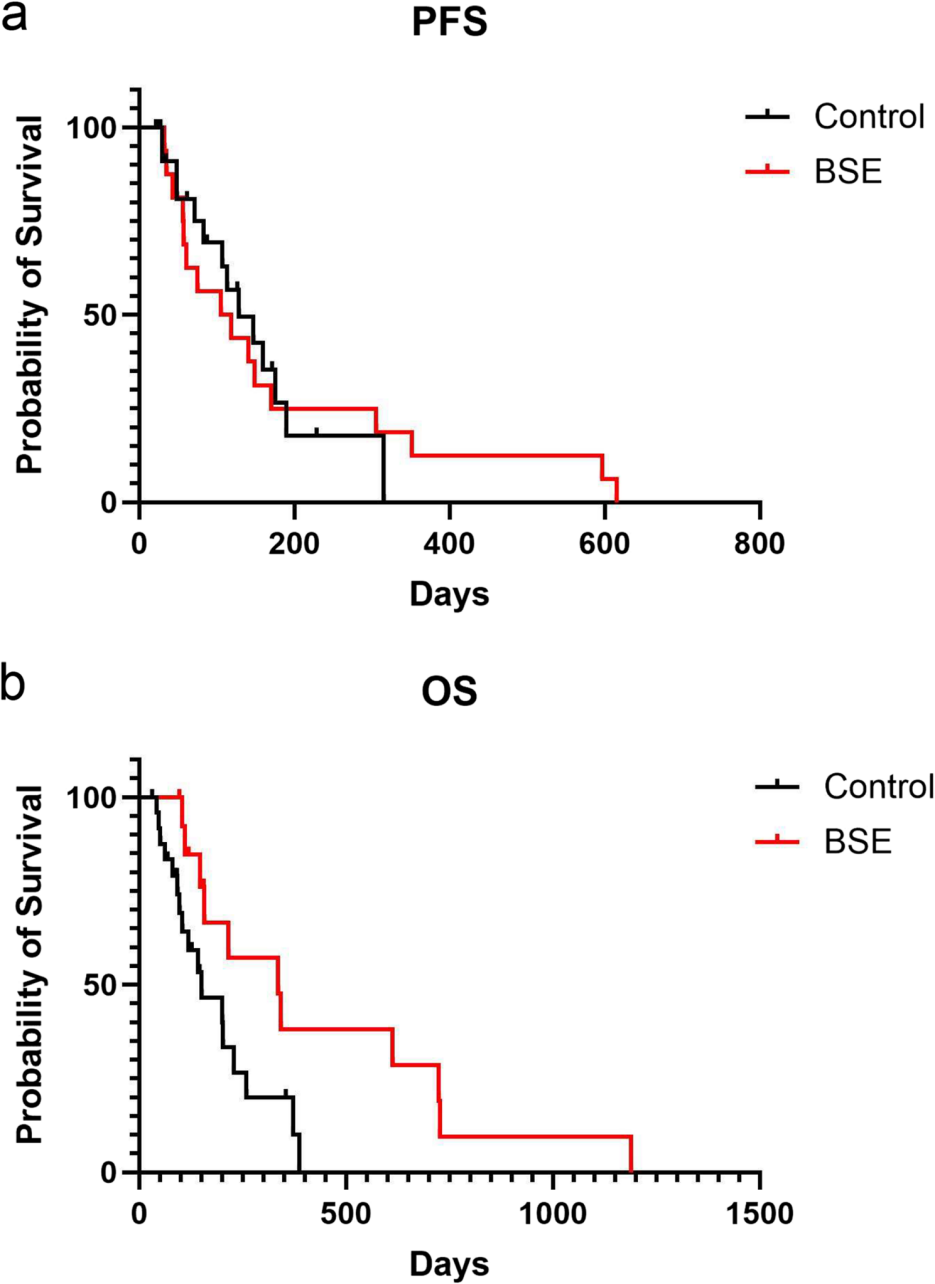
Clinical efficacies of BSE in the phase 2 study. The PFS of BSE and control groups did not differ significantly (**a**). The OS of both groups differed (**b**). Black line indicates control group and red line indicates BSE group

No objective responses were observed by BSE administration in the 14 cases with macroscopic lesions (data not shown). On the other hand, recurrence was not observed in the cases without macroscopic lesions (#3 and 5 cases). However, PFS was 111.5 (range, 32–615 days) for the BSE group and 128 days (range, 22–315 days) for the control group. OS was 335 days for the BSE group (range, 96–1189 days) and 150 days for the control group (range, 30–389 days). The OS of the BSE and control groups differed significantly (*p* = 0.019), but no significant difference in PFS was observed (*p* = 0.780, Fig. 3). To avoid the impact of difference of therapeutic modality on survival, the survival differences between BSE excluding the dogs treated with surgical resection and control groups were also assessed. As a result, the OS of BSE group was significantly longer than that of control group (342 days vs. 150 days, *p* = 0.0394). The factors associated with treatment response were not determined by univariate analysis (Table 5).

**Table 5:** Univariable analysis of factors in Table 2 assessed for an association with PFS and OS

	PFS		OS	
	HR (95%CI)	*p-value*	HR (95%CI)	
Age	0.952 (0.704-1.287)	0.749	1.038 (0.756-1.424)	0.819
B. W.	1.295 (0.958-1.751)	0.100	1.654 (0.982-2.786)	0.059
Primary lesion	0.608 (0.273-1.354)	0.223	0.510 (0.176-1.481)	0.216
Lymphnode metastasis	0.577 (0.179-1.865)	0.359	0.563 (0.148-2.149)	0.400
Bone lysis	1.891 (0.527-6.764)	0.328	1.228 (0.312-4.839)	0.769
Starting dose	1.002 (0.994-1.011)	0.615	0.999 (0.988-1.010)	0.855
Primary treatment	1.968 (0.543-7.125)	0.303	0.708 (0.166-3.026)	0.641
ALC	1.080 (0.367-3.184)	0.888	0.994 (0.270-3.662)	0.993

*B.W* Body weight, *ALC* Adequate local control, *PFS* Progression free survival, *OS* Overall survival, *HR* Hazard ratio, *CI* Confidence interval

## Discussion

This study revealed that BSE administration at 200 mg/kg is well tolerated and appears to contribute to prolonged OS in dogs with stage 3 oral melanoma. BSE group included the dogs treated with surgical resection, although control group did not include ones. Therefore, we assessed OS of BSE group excluding the dogs with surgical resection, and the OS was not affected by therapeutic modality for primary lesions. In addition, the OS of BSE group was numerically longer than that of dogs treated with surgical treatment (207 days) and 3D-CRT (163 days) previously reported [2, 27]. These findings suggest that BSE contributes to prolonged OS regardless of the kinds of local therapy.

However, BSE did not affect the PFS of the patients with melanoma after local therapy. This result may be due to a type 2 error, as PFS for 3 of the 16 dogs in the BSE group was longer than the range of the control group. The average PFS for the BSE group (181.6 days) was also longer than that of the control group (93.7 days). Six of 16 dogs (37.5%) survived numerically longer than the previously reported median OS for stage 3 melanoma.

BSE contained petasin, neopetasin, S-neopetasin, and S-petasin as petasin derivatives. These derivatives were considered to contribute to the clinical efficiency because similar anti-proliferative effects were observed among them in previous in vitro experiments [23]. However, detail pharmacokinetics (PK) in dogs and the mechanisms underlying the clinical efficacy of the petasin derivatives remain unclear. Although maximum plasma level of orally administered BSE had been evaluated in our preliminary experiment using a healthy beagle dog, PK might be various among the dogs with oral melanoma, leading to the difference of clinical efficacy. Another hypothesis was that petasin may not have a direct cytotoxic effect on melanoma cells, because the reduction of macroscopic lesions was not observed and no significant difference in PFS was observed between the groups. Previous in vitro studies have reported that petasin inhibits ETCC1, which plays a pivotal role in the glycolytic pathway and TCA cycle involved in cancer-specific metabolism [23, 28, 29]. This effect may influence cancer cell growth, but not cancer cell death. However, higher doses of BSE may have cytotoxic effects. To validate the more detail mechanisms of clinical efficiencies, PK and the activity of cytotoxic T cell and metabolome analysis need to be validated in dogs administered BSE.

This study had limitations. PK analysis was limited. In addition, the MTD for BSE was not determined because of the excessive number of tablets required for oral melanoma. Actually, the dog weighted 5 kg, which was almost median body weight of the dogs enrolled in the phase 2 trial, needed to be orally administered five 10 mm diameter tablets a day. An enrichment technique should be developed in the future, and decision of MTD of BSE based on AE and PK data and the evaluation of clinical efficiencies of MTD are needed. And, historical cases were adopted as controls, which might have the potential selection bias, although the supportive care was not different between BSE and control group. In the future, a randomized double blind phase 3 trial will be performed.

## Conclusions

BSE at a dose of 200 mg/kg was safely administered, and it helped prolong OS in dogs with oral melanoma when compared to historical controls. It is a promising therapeutic agent for canine oral melanoma.

## Supplementary Information

Supplementary Material 1.

## Data Availability

All data analysed during this study are included in this published article.

## Abbreviations

AEs: Adverse events
ALC: Adequate local control
ALT: Alanine aminotransferase
BSE: Butterbur shoot extract
CT: Computed tomography
ETCC1: Electron transport chain complex 1
MTD: Maximum tolerated dose
OS: Overall survival
PFS: Progression-free survival
3D-CRT: Three-dimensional conformal radiation therapy

## References

[1] Bergman PJ, Selmic LE, Kent MS. Melanoma. In: Vaill DM, Thamm DH, Liptak JM, editors. Withrow & macewen’s small animal clinical oncology. 6th ed. St Louis: Elsevier; 2020. pp. 367–81.

[2] Tuohy JL, Selmic LE, Worley DR, Ehrhart NP, Withrow SJ. Outcome following curative-intent surgery for oral melanoma in dogs: 70 cases (1998–2011). J Am Vet Med Assoc. 2014;245:1266–73. 25406707 10.2460/javma.245.11.1266

[3] Bergman PJ, A. C-PM, McKnight JA, Leibman NF, Craft DM, Leung C, Liao J, Riviere I, Sadelain M, Hohenhaus AE et al. Development of a xenogeneic DNA vaccine program for canine malignant melanoma at the Animal Medical Center. Vaccine : 2006;24:4582–4585. 10.1016/j.vaccine.2005.08.027 16188351

[4] Boston SE, Lu X, Culp WTN, Montinaro V, Romanelli G, Dudley RM, Liptak JM, Mestrinho LA, Buracco P. Efficacy of systemic adjuvant therapies administered to dogs after excision of oral malignant melanomas: 151 cases (2001–2012). J Am Vet Med Assoc. 2014;245:401–7. 25075823 10.2460/javma.245.4.401

[5] Grosenbaugh DA, Leard AT, Bergman PJ, Klein MK, Meleo K, Susaneck S, Hess PR, Jankowski MK, Jones PD, Leibman NF, et al. Safety and efficacy of a xenogeneic DNA vaccine encoding for human tyrosinase as adjunctive treatment for oral malignant melanoma in dogs following surgical excision of the primary tumor. Am J Vet Res. 2011;72:1631–8. 22126691 10.2460/ajvr.72.12.1631

[6] McLean JL, Lobetti RG. Use of the melanoma vaccine in 38 dogs: the South African experience. J S Afr Vet Assoc. 2015;86:1246. 26016668 10.4102/jsava.v86i1.1246 PMC6138178

[7] Ottnod JM, Smedley RC, Walshaw R, Hauptman JG, Kiupel M, Obradovich JE. A retrospective analysis of the efficacy of oncept vaccine for the adjunct treatment of canine oral malignant melanoma. Vet Comp Oncol. 2013;11:219–29. 23909996 10.1111/vco.12057

[8] Treggiari E, Grant JP, North SM. A retrospective review of outcome and survival following surgery and adjuvant xenogeneic DNA vaccination in 32 dogs with oral malignant melanoma. J Vet Med Sci. 2016;78:845–50. 26781703 10.1292/jvms.15-0510 PMC4905841

[9] Turek M, LaDue T, Looper J, Nagata K, Shiomitsu K, Keyerleber M, Buchholz J, Gieger T, Hetzel S. Multimodality treatment including ONCEPT for canine oral melanoma: A retrospective analysis of 131 dogs. Vet Radiol Ultrasound. 2020;61:471–80. 32323424 10.1111/vru.12860

[10] Verganti S, Berlato D, Blackwood L, Amores-Fuster I, Polton GA, Elders R, Doyle R, Taylor A, Murphy S. Use of oncept melanoma vaccine in 69 canine oral malignant melanomas in the UK. J Small Anim Pract. 2017;58:10–6. 28094857 10.1111/jsap.12613

[11] Giuliano A, Pimentel PAB, Horta RS. Checkpoint Inhibitors in Dogs: Are We There Yet? Cancers (Basel). 2024;16:2003. 10.3390/cancers16112003 PMC11171034 38893123

[12] Igase M, Inanaga S, Tani K, Nakaichi M, Sakai Y, Sakurai M, Kato M, Tsukui T, Mizuno T. Long-term survival of dogs with stage 4 oral malignant melanoma treated with anti-canine PD-1 therapeutic antibody: A follow-up case report. Vet Comp Oncol. 2022;20:901–5. 35535636 10.1111/vco.12829

[13] Igase M, Nemoto Y, Itamoto K, Tani K, Nakaichi M, Sakurai M, Sakai Y, Noguchi S, Kato M, Tsukui T, et al. A pilot clinical study of the therapeutic antibody against canine PD-1 for advanced spontaneous cancers in dogs. Sci Rep. 2020;10:18311. 33110170 10.1038/s41598-020-75533-4 PMC7591904

[14] Maekawa N, Konnai S, Nishimura M, Kagawa Y, Takagi S, Hosoya K, Ohta H, Kim S, Okagawa T, Izumi Y, et al. PD-L1 immunohistochemistry for canine cancers and clinical benefit of anti-PD-L1 antibody in dogs with pulmonary metastatic oral malignant melanoma. NPJ Precis Oncol. 2021;5:10. 33580183 10.1038/s41698-021-00147-6 PMC7881100

[15] Maekawa N, Konnai S, Takagi S, Kagawa Y, Okagawa T, Nishimori A, Ikebuchi R, Izumi Y, Deguchi T, Nakajima C, et al. A canine chimeric monoclonal antibody targeting PD-L1 and its clinical efficacy in canine oral malignant melanoma or undifferentiated sarcoma. Sci Rep. 2017;7:8951. 28827658 10.1038/s41598-017-09444-2 PMC5567082

[16] Kulinowski Ł, Luca SV, Minceva M, Skalicka-Woźniak K. A review on the ethnobotany, phytochemistry, Pharmacology and toxicology of butterbur species (Petasites L). J Ethnopharmacol. 2022;293:115263. 35427728 10.1016/j.jep.2022.115263

[17] Oelkers-Ax R, Leins A, Parzer P, Hillecke T, Bolay HV, Fischer J, Bender S, Hermanns U, Resch F. Butterbur root extract and music therapy in the prevention of childhood migraine: an explorative study. Eur J Pain. 2008;12:301–13. 17659990 10.1016/j.ejpain.2007.06.003

[18] Käufeler R, Polasek W, Brattström A, Koetter U. Efficacy and safety of butterbur herbal extract Ze 339 in seasonal allergic rhinitis: postmarketing surveillance study. Adv Ther. 2006;23:373–84. 16751170 10.1007/BF02850143

[19] Guo L, Kang JS, Kang NJ, Choi YW. S-petasin induces apoptosis and inhibits cell migration through activation of p53 pathway signaling in melanoma B16F10 cells and A375 cells. Arch Biochem Biophys. 2020;692:108519. 32763235 10.1016/j.abb.2020.108519

[20] Guo L, Kang JS, Park YH, Je BI, Lee YJ, Kang NJ, Park SY, Hwang DY, Choi YW. S-petasin inhibits lipid accumulation in oleic acid-induced HepG2 cells through activation of the AMPK signaling pathway. Food Funct. 2020;11:5664–73. 32542253 10.1039/d0fo00594k

[21] Guo L, Li K, CZ W, KJ S, SB G, Choi YW. S-Petasin isolated from petasites japonicus exerts anti-adipogenic activity in the 3T3-L1 cell line by inhibiting PPAR-γ pathway signaling. Food Funct. 2019;10:4396–406. 31282906 10.1039/c9fo00549h

[22] Lyu X, Song AL, Bai YL, Xu XD, He DQ, Zhang YC. Inhibitory effects of Petasin on human colon carcinoma cells mediated by inactivation of Akt/mTOR pathway. Clin Med J (Engl). 2019;132:1071–8. 10.1097/CM9.0000000000000199 PMC6595872 30896562

[23] Heishima K, Sugito N, Soga T, Nishikawa M, Ito Y, Honda R, Kuranaga Y, Sakai H, Ito R, Nakagawa T, et al. Petasin potently inhibits mitochondrial complex I-based metabolism that supports tumor growth and metastasis. J Clin Invest. 2021;131:e139933. 34623325 10.1172/JCI139933 PMC8409585

[24] Percie Du Sert N, Hurst V, Ahluwalia A, Alam S, Avey MT, Baker M, Browne WJ, Clark A, Cuthill IC, Dirnagl U, et al. The ARRIVE guidelines 2.0: updated guidelines for reporting animal research. PLoS Biol. 2020;18:e3000410. 32663219 10.1371/journal.pbio.3000410 PMC7360023

[25] LeBlanc AK, Atherton M, Bentley RT, Boudreau CE, Burton JH, Curran KM, Dow S, Giuffrida MA, Kellihan HB, Mason NJ, et al. Veterinary cooperative oncology Group-Common terminology criteria for adverse events (VCOG-CTCAE v2) following investigational therapy in dogs and cats. Vet Comp Oncol. 2021;19:311–52. 33427378 10.1111/vco.12677 PMC8248125

[26] Nguyen SM, Thamm DH, Vail DM, London CA. Response evaluation criteria for solid tumours in dogs (v1.0): a veterinary cooperative oncology group (VCOG) consensus document. Vet Comp Oncol. 2015;13:176–83. 23534501 10.1111/vco.12032

[27] Kawabe M, Mori T, Ito Y, Murakami M, Sakai H, Yanai T, Maruo K. Outcomes of dogs undergoing radiotherapy for treatment of oral malignant melanoma: 111 cases (2006–2012). J Am Vet Med Assoc. 2015;247:1146–53. 26517618 10.2460/javma.247.10.1146

[28] Lunt SY, Vander Heiden MG. Aerobic glycolysis: meeting the metabolic requirements of cell proliferation. Annu Rev Cell Dev Biol. 2011;27:441–64. 21985671 10.1146/annurev-cellbio-092910-154237

[29] Vander Heiden MG, DeBerardinis RJ. Understanding the intersections between metabolism and cancer biology. Cell. 2017;168:657–69. 28187287 10.1016/j.cell.2016.12.039 PMC5329766

