# Supplementary material for: Clinical impact of butterbur shoot extract in dogs with oral melanoma: a combined phase 1 and 2 clinical trial
Source: BMC Vet Res. 2026 Feb 20;22:191. doi: 10.1186/s12917-026-05326-w (PMC13032699; doi:10.1186/s12917-026-05326-w)
Supplement: Supplementary file 1 — Supplementary Material 1. [file 12917_2026_5326_MOESM1_ESM.pdf]

Additional file 1. Stability of BSE tablets in 25, 30, and 40 ℃.

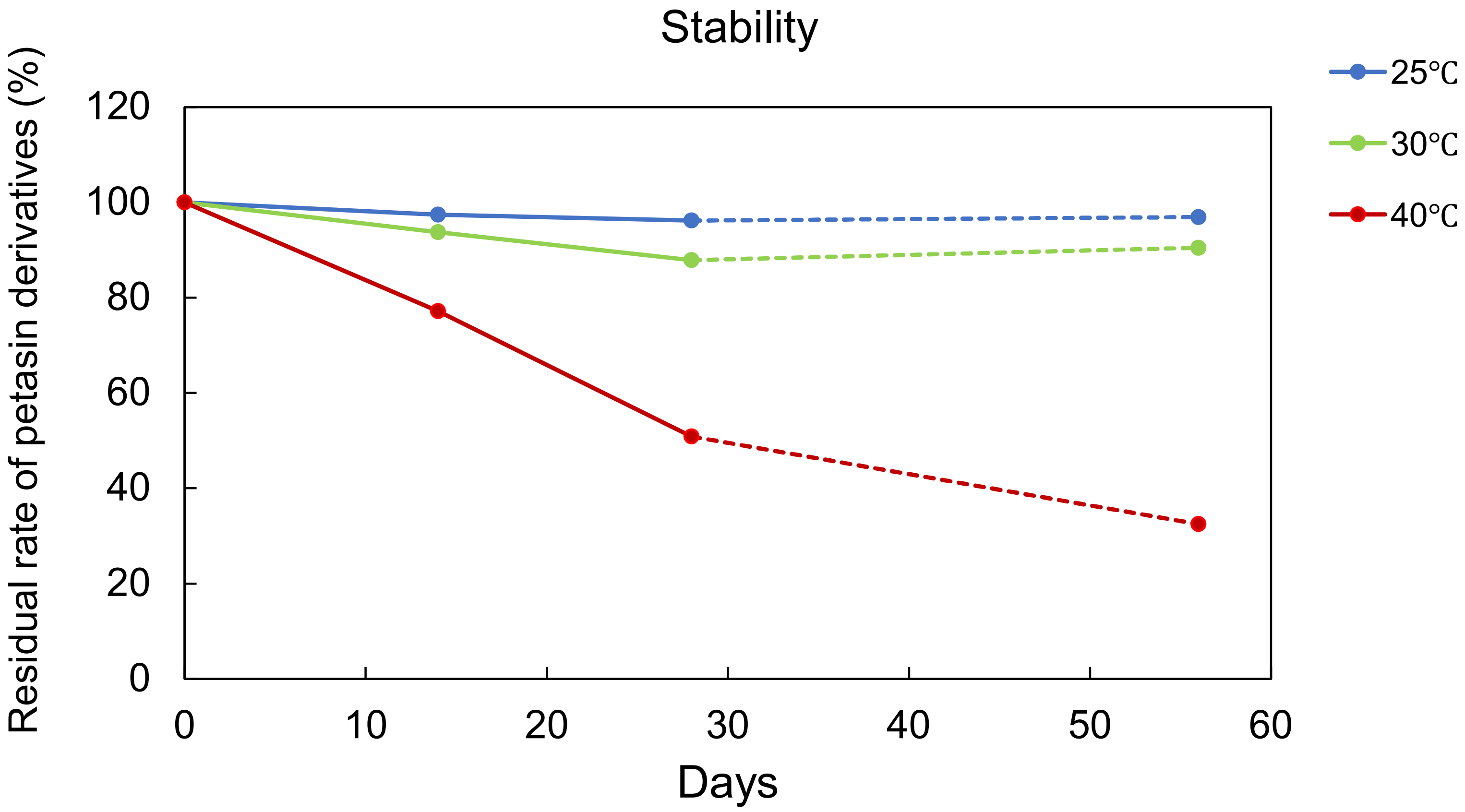

Residual rate (%)

Temperature (℃)	Retention period (days) 0	14	28
25	100	97.4	96.2
30	100	93.7	87.9
40	100	77.2	50.8

Petasin derivatives were detected by HPLC-PDA method after BSE tablets were resolved in Dimethyl Sulfoxide.
